# Examining the complex relationship between Urbanization and ecological environment in ecologically fragile areas: a case study in Southwest China

**DOI:** 10.3389/fpubh.2024.1358051

**Published:** 2024-05-16

**Authors:** Lei Liu, Yimeng Guo, Yuchao Li, Lanyue Zhang

**Affiliations:** ^1^Chengdu University of Technology, Chengdu, China; ^2^Neijiang Normal University, Neijiang, China; ^3^Sichuan Institute of Administration, Chengdu, China; ^4^Sichuan University Jinjiang College, Meishan, Sichuan, China

**Keywords:** urbanization, ecological environment, coupling coordination degree, obstacle factors model, Southwest China

## Abstract

The sustainable development of ecologically fragile areas and the implementation of regional coordinated development strategies cannot be separated from the coordinated development and common progress of urbanization and the ecological environment, and this is particularly the case in Southwest China. This study examines the interplay between urbanization and the ecological environment across 26 cities in Southwest China from 2009 to 2019, utilizing 30 statistical indicators to analyze their coupling coordination relationship and its spatiotemporal evolution. The Entropy TOPSIS method, the coupling coordination degree model, and the obstacle factors model were used to calculate the subsystem score, coupling coordination degree, and obstacle factors, respectively. Our findings reveal an upward trajectory in urbanization scores across the 26 cities, juxtaposed with a fluctuating downward trend in ecological environment scores. The coupling coordination degree of urbanization and ecological environment in most cities maintained a rapid upward trend and showed spatial distribution characteristics of “strong core, weak middle, and edge.” Moreover, our analysis identified public transport facilities, aggregate purchasing power, and cultural supply service services as primary obstacle factors impeding the development of coupling coordination degrees. These research results offer valuable insights for informing future endeavors in achieving high-quality development and fostering ecological civilization.

## 1 Introduction

Global sustainable development is currently confronted with a series of troubles, including over-exploitation, exhaustion of resources, degradation of the ecological environment, and pollution discharge (1). Traditional urbanization (UR) blindly pursues the speed of development, ignores the comprehensive benefits, and pays less attention to the external impact (2). Therefore, rapid urbanization is accompanied by unsatisfactory effects; there will inevitably be “urban diseases” such as resource scarcity, overpopulation, and a reduction in the quality of urban life (3–5), which will cause great pressure on the ecological environment (EE) and exceed its endurance range (6). In addition, the increasingly harsh EE also reversely limits the improvement of urbanization development efficiency and affects the further sustainable and healthy development of urbanization (7, 8). Accordingly, the mature development of UR needs a healthy EE as the carrier, while the change in EE restricts the quality and scale of urbanization development to some extent (9). UR and EE are significant contents of the man–land relationship system and are crucial topics for achieving sustainable development goals (10). The guarantee of the normal and stable progress of the region cannot be separated from the mutual coordination of their development pace. From “Garden City” in the 1890s (11) to “Man and Biosphere Programme” in the 2000s, the form of ecological urbanization is gradually pursued by people.

Urbanization refers to the transition of society from an agrarian way of life to a modern civilized way of life dominated by industry and service trades, with the continuous progress of productivity and the acceleration of industrialization (12). It is manifested as the spatial agglomeration of the population from the countryside to the city, accompanied by a change in the rural economy, culture, lifestyle, and other aspects of the city (13). The main factors influencing urbanization include urban–rural income gap and employment environment (14), government and institutional factors (15), and the spillover effect of big cities (16). The ecological environment is the general term for the living environment and abiotic environment, which is firmly connected with human wellbeing, health, and human settlement safety (17). Environmental degradation and ecological problems will negatively affect the health of the population (18). The main factors affecting the ecosystem include population activity, progress in urbanization, and climate change (19, 20). Coupling is an interaction mechanism between two or more systems (21, 22), which has been broadly applied in the fields of natural science, social science, and humanities. Academia has carried out a lot of exploration of the interactive relationship between UR and EE.

According to different research objects, scholars have analyzed the coupling coordination degree (CCD) of UR and EE from different angles and promoted theoretical innovation, methodical innovation, and practical innovation based on the rich research in existence. The CCD studies of UR and EE are mainly supported by the Environmental Kuznets Curve (23), the theory of urban metabolism (24), and the theory of ecocity (25). There is copious research on the CCD of UR and EE, with much of it focusing on the relationship between UR and individual ecological indicators. For example, studies have explored the coupling characteristics of economic benefit and ecological management in various cities and regions (26), as well as the influence of urbanization on agricultural development (27). Most empirical conclusions revealed that UR has more negative than positive impacts on EE (28). The definition of UR and EE CCD is constantly improving, and the innovation and improvement of new methods and new models are constantly being progressed. At present, there are PSR (29), the coupling coordination degree model (30), the multi-agent model (31), the integrated response model (32), and the PCA model (33), which are the major methods of evaluation. The mainstream methods to determine the index system weight include the entropy weight method and the analytic hierarchy process (34). Dynamic development and trend prediction analysis are also important research directions of CCD of UR and EE, and scholars mainly use spectral analysis (35), the deviation ellipse model (36), and the gray forecast model (37) to conduct relevant empirical tests.

By the end of 2021, according to the resident population of China, the rate of UR was 64.72%, an increase of about 2.6 times from 17.92% in 1978, which is an unprecedented growth rate in the world (38). Urbanization, as a great source of driving force for social progress and improving the standard of living, has also brought about plenty of resources and environmental hazards, including water resource depletion, air pollution, and destruction of ecological diversity, affecting economic transformation and upgrading (39). China is standing at a critical node that promotes the urbanization development model to adapt to the transformation of the economic situation for now, aiming to integrate harmony, ecology, and livable development into urbanization (40). Temporary development at the cost of ecological risks goes against the human-centered principle.

The interaction between UR and EE in the southwest region is prominent. Since the implementation of the Western Development Strategy, the southwest region has become an important growth pole for China’s economic development, relying on abundant labor resources and unique geographical advantages and supported by policies such as the “Belt and Road” and the “China–Myanmar–Bangladesh–India Economic Corridor.” The rapid expansion of cities has highlighted the imbalance in urban–rural development. Therefore, coordinating the development of new-type urbanization and promoting deep integration of urban and rural domains stands as a critical imperative for the future progression of Southwest China. In general, the level of social productivity is lower than that of other regions, the economic base is still very weak, and it is still in the process of expanding urban capacity and improving urban quality (41).

Meanwhile, the southwest region is an important ecological and environmental protection barrier in China, with prominent ecological vulnerability issues. The area exposes extensive karst stratum, leading to poor soil fertility and rapid drainage of rainfall (42). The lack of soil fertility and water resources caused by the above reasons makes the natural ecology of the southwest region extremely fragile (43). Furthermore, with the speeding up of UR, heavy industry has become an essential driving force to boost economic growth, and the pressure on the ecosystem will increase, which will threaten the biodiversity of the ecosystem in the short term so that the cities in Southwest China will show the trend of urbanization and ecological environment imbalance (44). Attaching importance to the harmonious relationship between UR and EE in Southwest China and getting rid of the traditional industrial operation mode of energy-intensive, heavy pollution, and low efficiency can provide favorable help for the less developed areas to improve their urban competitiveness and realize the green rise.

**Figure 1:**
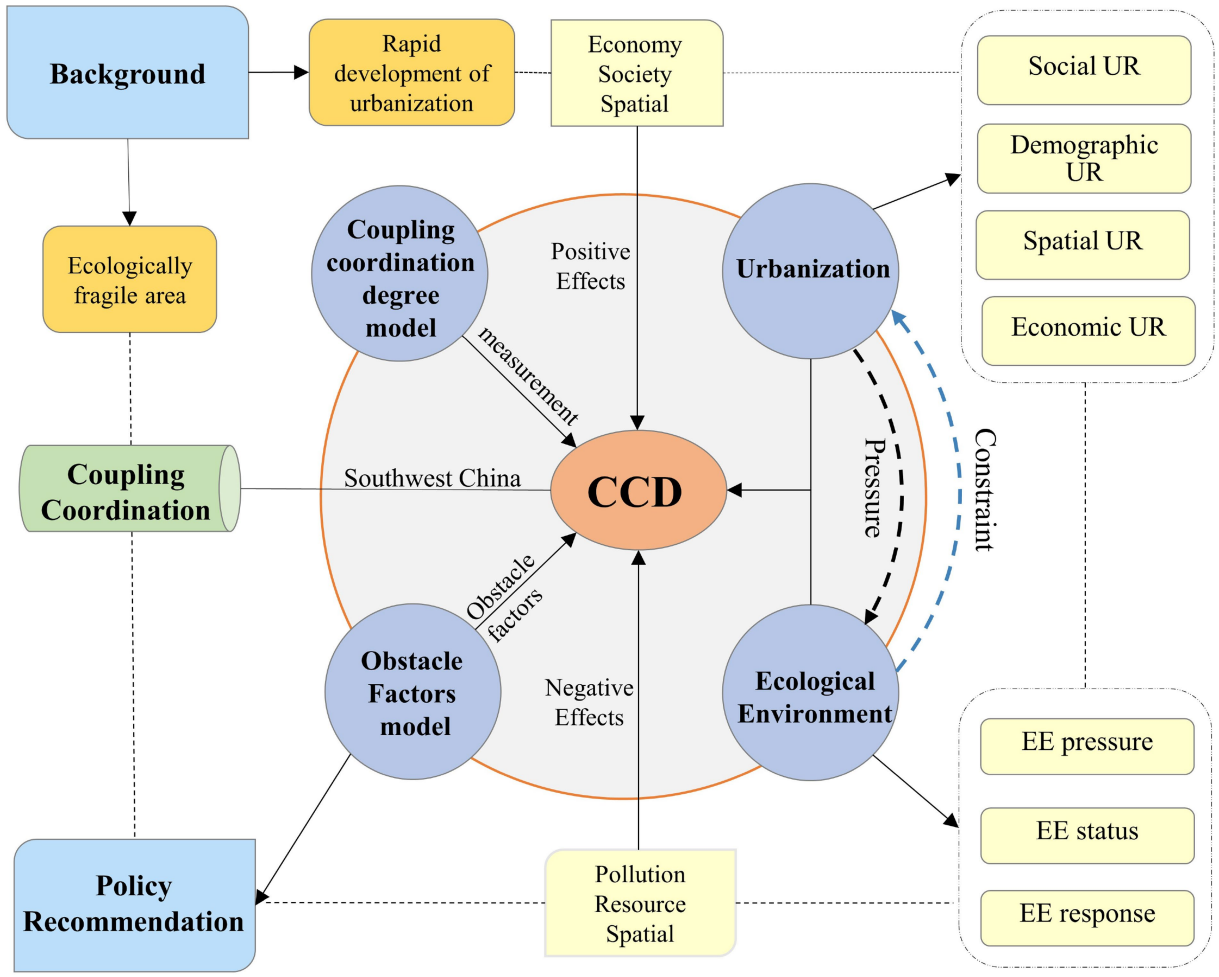
Theoretical framework of this study.

The logical structure of this article is shown in Figure 1. In the background of ecological vulnerability and the rapid development of urbanization, 26 cities in Southwest China are the objects of study. The four levels of demographic, economy, space, and society are the selection of UR index and EE evaluation indicators from three aspects: EE state, EE pressure, and EE response. Based on the evaluation index system, the scores of the two systems were measured separately using the entropy weight TOPSIS method. Furthermore, the CCD model was employed to analyze the evolution process and law of the CCD of EE and UR. Finally, the obstacle factors affecting UR development and EE quality are identified by the obstacle factor model. Specific and feasible policy suggestions are proposed by combining the results of obstacle factor analysis and reality.

To coordinate the complex interactive relationship between UR and EE, this study takes 26 cities in Southwest China as the research object to carry out the analysis. This article will conduct further research from two aspects. On the one hand, in the evaluation of the CCD of UR and EE in Southwest China, multiple dimensions are included to identify the obstacle factors of the CCD, making the evaluation results of it and its restrictive factors more comprehensive, reasonable, and objective. On the other hand, taking the 26 representative cities of Southwest China as a case study, the spatial–temporal evolution trends of UR, EE, and CCD of both are analyzed for the sake of providing policy recommendations for green and sustainable development in ecologically weak areas and then balancing regional development differences.

## 2 Research methods and data sources

### 2.1 Study area

**Figure 2:**
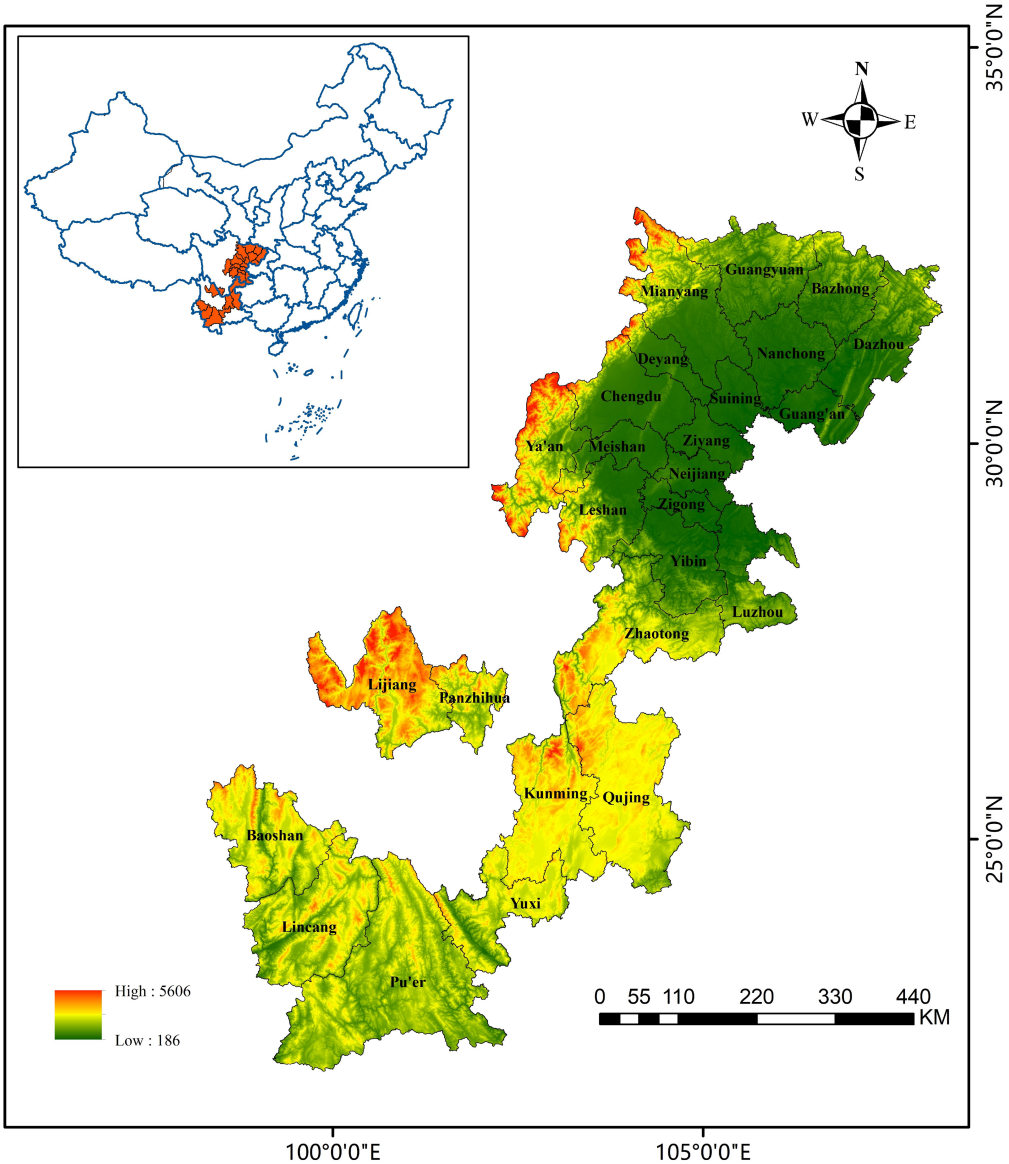
The spatial location of the cities in the Southwest China region.

Southwest China, with its vast territory and sparse population, is one of the earliest agricultural areas in China. It has created a splendid culture and promoted the progress of human civilization. It is not only the water conservation area of the middle and lower reaches of the Yangtze River but also the key “source” of ecological balance. However, man-made destruction of vegetation and irrational use of land resources result in barren land, rocky desertification, and frequent disasters. Chongqing, being a provincial-level municipality, does not fit within the same classification as other urban areas, thus rendering comparisons inappropriate. Consequently, the study area does not encompass Chongqing Municipality. Furthermore, given the presence of numerous minority autonomous regions and cities within the Southwest region, the data published by these areas may lack comprehensiveness, resulting in numerous instances of missing data values. Therefore, to uphold the study’s robustness, regions with incomplete data are excluded. Taking these factors into account, 26 cities, excluding Chongqing, were included in this study (Figure 2). The empirical study of the CCD of UR and EE in 26 cities is of valuable significance for improving regional ecological conditions, exploring economic potential, and fostering harmonious social life.

### 2.2 Index system construction

Research on the CCD of UR and EE systems is extensive, primarily focusing on the relationship between individual UR indicators and single EE indicators. Notable among these are eco-efficiency indicators such as carbon emission (45), resource status (46), water resources (47), and the Normalized Difference Vegetation Index [NDVI (48);]. However, the ecological environment is characterized by complex and diverse ecosystems, making reliance on a single indicator insufficient for fully assessing ecological quality. Recognizing this limitation, to precisely and all-sided present the comprehensive traits of ecosystems (49), scholars have built many kinds of ecological environment evaluation index systems, including physiological conditions and natural geography, which provide a basis and guarantee for ecosystem health evaluation, ecological protection and restoration, ecological compensation, and ecological carrying capacity evaluation (50, 51).

Furthermore, given the dynamic nature of social and economic development, urbanization represents a multifaceted integration of the social environment, economic status, land use, and population agglomeration. Recognizing that the urbanization of a single population cannot fully represent the level of urbanization, scholars began to consider using more comprehensive indicators (52, 53).

UR and EE systems both contain a variety of dimensions and many influencing factors, which belong to the non-linear complex system. After the interaction and coupling of the two, a more complicated system is formed. Therefore, referring to the frequency of various indicators in existing studies, in line with the principles of comprehensiveness and availability, and combined with the development characteristics of 26 cities, the evaluation index system of UR and EE in Southwest China was established. As shown in Table 1, the positive indicator means that the indicator has a positive promoting effect on urbanization development and ecological environment level, while the negative indicator has a negative inhibiting effect.

**Table 1:** Indicator systems of UR and EE (2009–2019).

Elements	Primary indicator	Indicators	Attribute
Urbanization	Demographic urbanization	X_1_ Urbanization rate (%)	+
		X_2_ Registered unemployed persons in urban areas (person)	−
		X_3_ Proportion of secondary sector of the economy employed (%)	+
		X_4_ Proportion of tertiary sector of the economy employed (%)	+
	Economic urbanization	X_5_ *Per capita* GDP (yuan)	+
		X_6_ Secondary and tertiary industry as a percentage to GDP (%)	+
		X_7_ Total retail sales of consumer goods (10,000 yuan)	+
		X_8_ Average wage of employed staff and workers (yuan)	+
		X_9_ Household saving deposits at year-end (10,000 yuan)	+
	Spatial urbanization	X_10_ Built-up area (sq. km)	+
		X_11_ Area of land used for L living (sq. km)	+
		X_12_ Land used for urban construction as a percentage of urban area (%)	+
		X_13_ Area of the city paved roads at year-end (10,000 sq. m)	+
	Social urbanization	X_14_ Number of buses and trolley buses under operation at year-end (unit)	+
		X_15_ Revenue from postal and telecommunication services (10,000 yuan)	+
		X_16_ Number of beds of hospitals (bed)	+
		X_17_ Collections of public libraries (1,000 copies)	+
		X_18_ Number of subscribers of mobile telephones at year-end (10,000 households)	+
		X_19_ Number of subscribers of internet services (10,000 households)	+
		X_20_ The education expenditure accounts for the proportion of the local general public budget expenditure (%)	+
Ecological environment	Ecological environment pressure	X_21_ Volume of industrial wastewater discharged (10,000 tons)	−
		X_22_ Volume of industrial sulfur dioxide emission (ton)	−
		X_23_ Volume of industrial soot (dust) emission (ton)	−
	Ecological environment status	X_24_ The proportion of green land (%)	+
		X_25_ The proportion of parks green land (%)	+
		X_26_ Green coverage of built-up areas (%)	+
		X_27_ Industrial electricity consumption (10,000 kWh)	−
	Ecological environment response	X_28_ Ratio of consumption wastes treated (%)	+
		X_29_ Ratio of wastewater centralized treated of sewage work (%)	+
		X_30_ Ratio of industrial solid wastes comprehensively utilized (%)	+

### 2.3 Data sources and methodology

#### 2.3.1 Data sources

This study uses the annual data from 2009 to 2019 for UR and EE of 26 cities in Southwest China. The economic and social data ranging from X1 to X30 are obtained from the China Urban Statistical Yearbook (2010–2020), the Sichuan Statistical Yearbook (2010–2020), and the Yunnan Statistical Yearbook (2010–2020). The individual missing values were supplemented by searching the Statistical Yearbook and Statistical Bulletin on National Economic and Social Development for the corresponding years of each province and city. For data with fewer missing years, data from adjacent years were supplemented using linear interpolation. Data with numerous missing years were deleted to ensure the reliability of the results.

#### 2.3.2 The entropy TOPSIS model

The entropy weight TOPSIS method amalgamates the strengths of both the entropy weight method and the TOPSIS method. The entropy weight method serves as an objective weighting approach, determining weights based on the information of each evaluation index. It not only reveals the importance of each index in the decision-making process and reflects the original information of each index but also depicts the changes in index weights over time, thus mitigating subjective bias to some extent and enhancing the scientific rigor of evaluation (54). On the other hand, TOPSIS evaluates the relative merits and demerits among finite objects. The entropy weight TOPSIS method initially calculates the objective weights of indicators using the entropy weight method and then applies the TOPSIS method to evaluate each object. This method’s objectivity mitigates bias stemming from subjective assignment and is suitable for the scientific evaluation of each subsystem. In this study, the index weight is determined by the entropy weight method, and then the TOPSIS method calculates the ideal solution to determine the ranking of the evaluation objects. The calculation steps are listed hereafter: (Equations 1–14)

(1) Construct the global evaluation matrix: Suppose there are m cities, n indicators, that constitute the initial matrix $C={\left(C_{\mathrm{ij}}\right)}_{m\times n}$ . The formula is as follows:

(1) $$C=\left[\begin{array}{ccc} C_{11} & \cdots & C_{1n}\\ \vdots & \ddots & \vdots \\ C_{m1} & \cdots & C_{mn} \end{array}\right]$$

(2) Data standardization processing:

The range method is used for standardization. The standardized formula for positive indicators is as follows:


(2) $$Z_{ij}=\frac{C_{ij}-\min\left(C_{ij}\right)}{\max\left(C_{ij}\right)-\min\left(C_{ij}\right)}+0.00001$$

The standardized formula for negative indicators is as follows:


(3) $$Z_{ij}=\frac{\max\left(C_{ij}\right)-C_{ij}}{\max\left(C_{ij}\right)-\min\left(C_{ij}\right)}+0.00001$$

In the formula, $Z_{ij}$ represents the standard value of the j index of the i evaluation object, $\max\left(C_{ij}\right)$ and $\min\left(C_{ij}\right)$ represent the maximum and minimum values of the original data, respectively. All $Z_{ij}$ values are in [0,1] after normalization.


(4) $$Z=\left[\begin{array}{ccc} Z_{11} & \cdots & Z_{1n}\\ \vdots & \ddots & \vdots \\ Z_{m1} & \cdots & Z_{mn} \end{array}\right]$$

(3) Calculate information entropy $E_{j}$ and information entropy redundancy $g_{j}$ :

(5) $$E_{j}=-k\sum_{m}^{i=1}P_{ij}\ln P_{ij}{,}_{1\leq i\leq m1\leq j\leq n}$$

Among them $P_{ij}=\frac{Z_{ij}}{\sum_{i=1}^{m}Z_{ij}},$ k=1/lnm, when $P_{ij}=0$ $Pij\ln P_{ij}=0$.


(6) $$g_{j}=1-E_{j}$$

(4) Define the weight of each indicator $w_{j}$ :

(7) $$w_{j}=\frac{g_{i}}{\sum_{j=1}^{n}g_{j}}$$

(5) Calculate the matrix of weights:

(8) $$R={\left(r_{ij}\right)}_{m\times n}$$

(9) $$r_{ij}=w_{j}\times Z_{ij}$$

(6) Determine the optimal and the worst solutions:

(10) $$s_{j}^{+}=\max\left(r_{1j},r_{2j},\cdots ,r_{mj}\right)$$

(11) $$s_{j}^{-}=\max\left(r_{1j},r_{2j},\cdots ,r_{mj}\right)$$

(7) Calculate the Euclidean distance between each scheme and the optimal, worst solution:

(12) $$sep_{i}^{+}=\sqrt{\overset{n}{\underset{j=1}{\sum }}{\left(s_{j}^{+}-r_{ij}\right)}^{2}}$$

(13) $$sep_{i}^{-}=\sqrt{\overset{n}{\underset{j=1}{\sum }}{\left(s_{j}^{-}-r_{ij}\right)}^{2}}$$

(8) Calculate the comprehensive rating index:

(14) $$V_{i}=\frac{sep_{i}^{-}}{sep_{i}^{+}+sep_{i}^{-}}\left(0\leq V_{i}\leq 1\right)$$

The value $V_{i}$ represents the advantages and disadvantages of a city’s comprehensive evaluation results in urbanization and EE in a certain year. The larger the value, the higher the evaluation. The stage division of UR and EE is shown in Table 2.

**Table 2:** Stage division of UR and EE.

Urbanization		Ecological environment	
Interval of UR score	UR stage	Interval of EE score	EE stage
0≤UR≤0.1	Level VI	0≤EE≤0.2	Level VI
0.1<UR≤0.2	Level V	0.2<EE≤0.3	Level V
0.2<UR≤0.4	Level IV	0.3<EE≤0.4	Level IV
0.4<UR≤0.6	Level III	0.4<EE≤0.6	Level III
0.6<UR≤0.8	Level II	0.6<EE≤0.8	Level II
0.8<UR≤1	Level I	0.8<EE≤1	Level I

#### 2.3.3 The CCD model

The CCD model serves as an analytical tool for assessing the level of coordinated development across various entities. It facilitates the examination of correlations, synergies, and coordination among different subsystems within a regional economic system. The coupling relationship between the UR system and the EE system is modeled through the CCD model as follows: (Equations 15–17)


(15) $$E=2{\left[\frac{U_{1}\times U_{2}}{{\left(U_{1}+U_{2}\right)}^{2}}\right]}^{1/2}$$

(16) $$D={\left(E\times T\right)}^{1/2}$$

(17) $$T=\alpha U_{1}+\beta U_{2}$$

$U_{1}$ represents the UR comprehensive score, and $U_{2}$ represents the EE comprehensive score. E represents the CD, D represents the CCD, and T represents the overall development index of UR and EE. α and β are specific weights, which are determined by the relative importance of urbanization and the EE system. This study suggests that the two are equally important in the UR–EE interaction coupling system, thus it is taken α=β=0.5 . According to the actual development of cities in Southwest China, the CCD of UR and EE is divided into five categories, as shown in Table 3.

**Table 3:** Stage division of coupling coordination degree between urbanization and EE.

Coordination degree value interval	Coordination stage
0≤D≤0.4	Maladjustment
0.4<D≤0.5	Barely coordination
0.5<D≤0.6	Primary coordination
0.6<D≤0.9	Intermediate coordination
0.9<D≤1	Good coordination

#### 2.3.4 The obstacle factors model

The obstacle factors model can calculate the obstacle degree of each evaluation index in the comprehensive evaluation, search for key factors that restrict the further development of things, clarify the factors that have the main influence on the evaluation results, and clarify the influence degree of the key constraints. In this study, the obstacle factors model is used to explore the main obstacle factors affecting the CCD of UR and EE for analysis and diagnosis (55). The formula is as follows: (Equation 18)


(18) $$Y_{jt}=\frac{\left(1-Z_{jt}\right)\times w_{j}\times 100\%}{\sum_{j=1}^{n}\left(1-Z_{jt}\right)\times w_{j}}$$

$w_{j}$ is the weight of the j index, and $Y_{jt}$ is the obstacle degree of the j index in t year.

**Figure 3:**
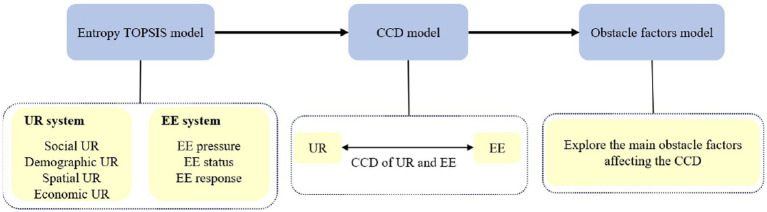
Methodological flowchart of this study.

Based on the preceding analysis, the methodological flowchart of this study is depicted in Figure 3.

## 3 Results

### 3.1 The analysis of the urbanization subsystem

The fluctuation of UR scores in 26 cities in Southwest China increased. According to the average UR score of each city, with a growth rate of 27.92%, Chengdu increased from 0.743 in 2009 to 0.951 in 2019, which is the highest quality of urbanization in Southwest China, and its score is much higher than other cities. In addition to Chengdu, Kunming is the only city whose average UR score is over 0.4. Most cities have UR scores hovering around 0.1. This shows that provincial capital cities with relatively prominent social and economic strength have the best effect on UR. From the perspective of the growth rate, four cities exceeded 50%, including Nanchong (64.32%), Ya’an (83.50%), Lijiang (59.75%), and Lincang (54.93%). Panzhihua (−5.71%), Deyang (−1.68%), and Kunming (−34.12%) show negative growth. The improvement of UR quality in 26 cities from 2009 to 2019 indicates that cities in Southwest China have achieved remarkable development in industrial transformation and upgrading, people’s living standards, and spatial pattern construction.

**Figure 4:**
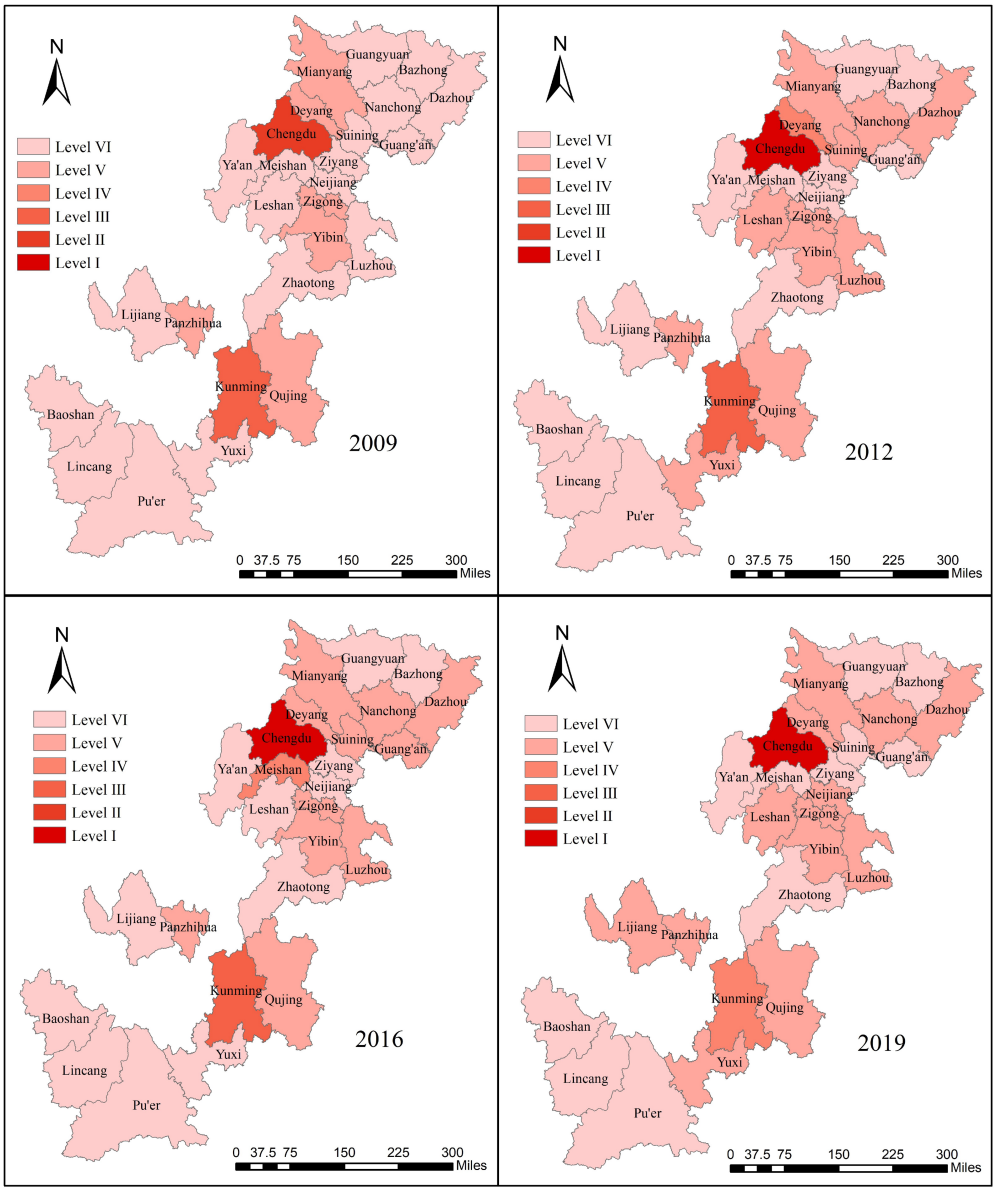
Spatial–temporal variation of score of UR.

The distribution of UR scores of 26 cities in Southwest China presents a significant feature of extreme spatial imbalance, showing the spatial characteristics of “strong core, weak middle, and edge” (Figure 4). Chengdu and Kunming, as the capital cities, have the best performance due to their strong economic strength. The level of urbanization in the remaining 24 cities is still in its infancy, rising gently, and most cities have improved but are ineffective. Specifically, from 2009 to 2012, eight cities achieved stage upgrading, while the remaining 18 cities remained unchanged. The level I cities grew from scratch due to the improvement of Chengdu, and the overall cities showed a significant improvement. From 2012 to 2016, two cities were upgraded, three cities were downgraded, and the remaining 21 cities remained unchanged, showing little change in the overall level. From 2016 to 2019, four cities were upgraded, four cities were downgraded, and the remaining 18 cities remained unchanged, showing a slight decline from 2016. The big difference in the UR scale among cities is mainly because of the difference in geographical location, distribution of population resources, and development strategies.

### 3.2 The analysis of the ecological environment subsystem

The fluctuation of EE scores in Southwest China decreased from 2009 to 2019. According to the average EE score of each city, Chengdu has the best EE quality in Southwest China, and its score rose from 0.769 in 2009 to 0.886 in 2019. Zhaotong has the worst EE quality in Southwest China, and its EE score decreased from 0.217 in 2009 to 0.141 in 2019, with a negative growth rate of 35.15%. In addition to Chengdu, six other cities scored above 0.25 on average, including Zigong (0.444), Deyang (0.264), Suining (0.409), Neijiang (0.254), Nanchong (0.256), and Kunming (0.351). Although most cities are non-resource-based, they still excel in the allocation of natural resources. In the field of growth rate, six cities show positive growth rates, namely, Chengdu (15.26%), Luzhou (34.86%), Neijiang (21.35%), Leshan (5.70%), Yibin (38.78%), and Dazhou (7.77%). The deterioration of EE in 20 cities from 2010 to 2019 indicates that cities in Southwest China have adopted unreasonable practices in the landscaping of the garden, natural resource utilization, and sewage treatment, resulting in an improvement in EE quality that is insignificant.

**Figure 5:**
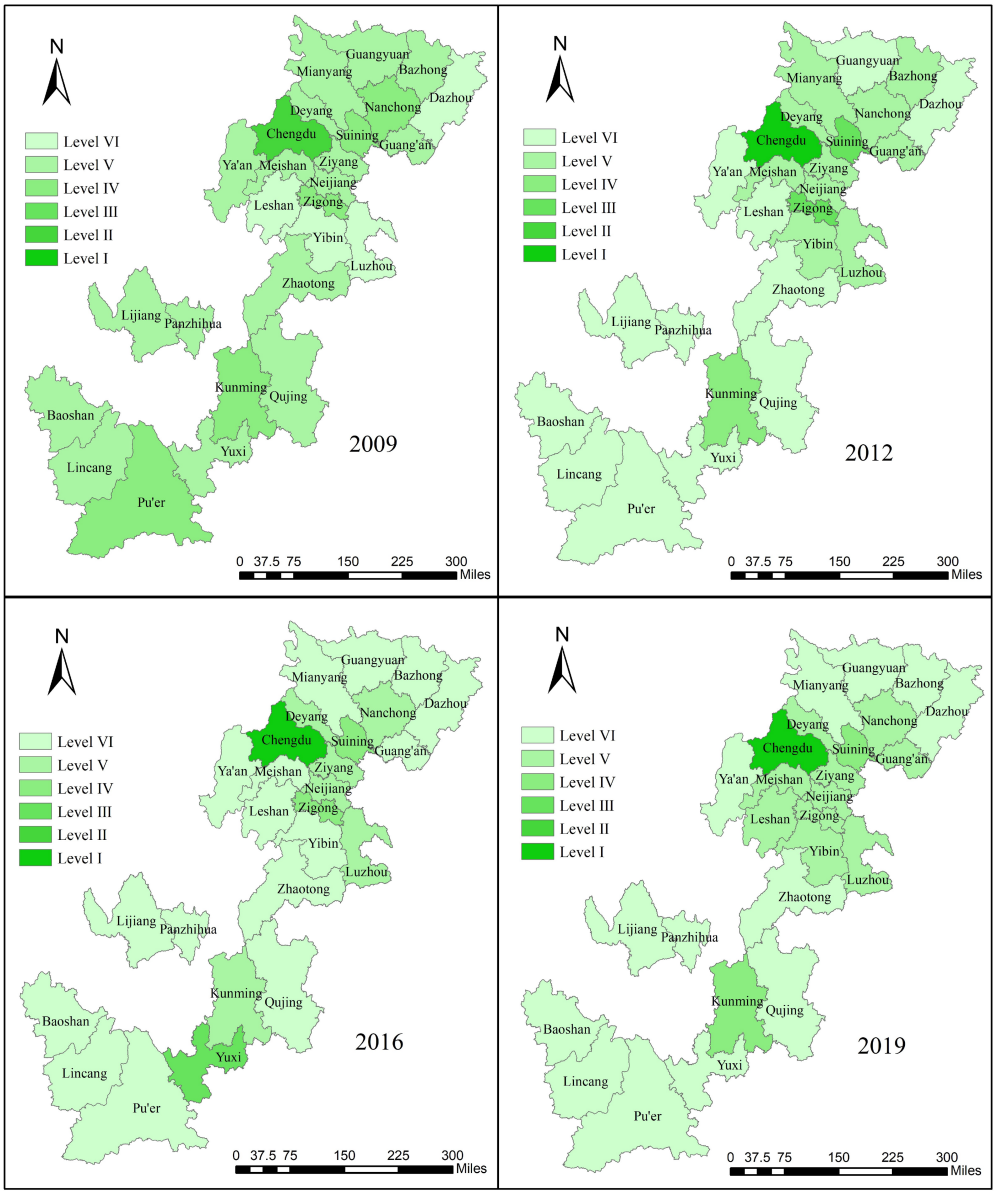
Spatial–temporal variation of score of EE.

According to the distribution of EE scores of 26 cities in Southwest China, it can be seen that the spatial distribution pattern of EE score is similar to that of UR score (Figure 5). The overall situation of EE quality development in various cities is not optimistic enough. Individual cities have made steady progress, and the upward trend is gentle. Specifically, from 2009 to 2012, five cities achieved stage upgrades, 10 cities were downgraded, and the remaining 11 cities remained unchanged. The overall level of level I cities increased due to Chengdu’s ecological environment protection from scratch. From 2012 to 2016, only Yuxi achieved stage upgrade; the other seven cities were downgraded; 18 cities remained unchanged; and the overall level decreased. From 2016 to 2019, four cities were upgraded, while one city was downgraded, and the remaining 21 cities remained unchanged, showing little change in the overall level. One of the reasons for this result is that the restoration of EE quality is a long-term process that requires more time and resources. Second, in the current economic situation, ecological compensation, purification of pollution, and expansion of greening are still seen as the “expend” and “sacrifice” of monetary benefits and have not received enough attention from the government and enterprises.

### 3.3 The analysis of the coupling coordination degree

The scores of the CCD of UR and EE in 26 cities of Southwest China were generally gradually upward from 2009 to 2019, between 0.298 and 0.958. The level of CCD in each city is distributed into five types, from imbalance to good coordination, with an overall improvement. In the field of the average CCD score from 2009 to 2019, Chengdu performed the best, increasing from 0.870 to 0.958 with a growth rate of 10.19%. Only Chengdu and Kunming have reached the good coordination and intermediate coordination stages, respectively, with average values of 0.933 and 0.637, and the other 24 cities are in the maladjustment stage and barely coordination stage. According to the growth rate of CCD from 2009 to 2019, Luzhou had the highest average annual growth rate of CCD, which was 17.55%. In addition to Luzhou, four other cities had a growth rate above 10%, including Chengdu (10.19%), Neijiang (15.52%), Yibin (14.08%), and Dazhou (11.29%). Negative growth rates in Kunming (−14.13%) and Qujing (−12.92%) exceed (10%) (Table 4).

**Table 4:** CCD of urbanization and EE.

City	2009	2010	2011	2012	2013	2014	2015	2016	2017	2018	2019	Average	Growth rate (%)
Chengdu	0.870	0.892	0.943	0.950	0.952	0.952	0.924	0.938	0.949	0.934	0.958	0.933	10.19
Zigong	0.441	0.471	0.498	0.508	0.529	0.519	0.497	0.477	0.485	0.485	0.424	0.485	−3.92
Panzhihua	0.425	0.423	0.431	0.422	0.455	0.427	0.413	0.395	0.398	0.390	0.385	0.415	−9.39
Luzhou	0.372	0.398	0.412	0.414	0.435	0.427	0.414	0.416	0.438	0.436	0.437	0.418	17.55
Deyang	0.434	0.432	0.436	0.500	0.447	0.442	0.426	0.406	0.431	0.408	0.416	0.434	−4.13
Mianyang	0.425	0.416	0.443	0.435	0.439	0.434	0.419	0.415	0.432	0.426	0.417	0.427	−2.03
Guangyuan	0.355	0.344	0.367	0.360	0.362	0.369	0.368	0.355	0.383	0.378	0.361	0.364	1.70
Suining	0.411	0.401	0.460	0.550	0.481	0.476	0.460	0.452	0.435	0.430	0.420	0.452	2.06
Neijiang	0.365	0.369	0.393	0.384	0.413	0.404	0.392	0.384	0.436	0.421	0.422	0.398	15.52
Leshan	0.366	0.370	0.370	0.378	0.382	0.371	0.355	0.348	0.371	0.364	0.378	0.368	3.32
Nanchong	0.414	0.418	0.421	0.437	0.440	0.434	0.425	0.419	0.429	0.422	0.444	0.428	7.20
Meishan	0.361	0.341	0.363	0.375	0.373	0.370	0.523	0.467	0.449	0.401	0.369	0.399	2.06
Yibin	0.358	0.375	0.406	0.403	0.403	0.387	0.383	0.372	0.388	0.404	0.409	0.390	14.08
Guang’an	0.352	0.386	0.394	0.393	0.383	0.384	0.382	0.377	0.403	0.385	0.373	0.383	5.97
Dazhou	0.345	0.377	0.362	0.366	0.370	0.375	0.351	0.354	0.383	0.368	0.384	0.367	11.29
Ya’an	0.336	0.337	0.316	0.353	0.348	0.351	0.349	0.336	0.359	0.359	0.352	0.345	4.78
Bazhong	0.341	0.333	0.356	0.342	0.353	0.354	0.351	0.348	0.347	0.329	0.343	0.345	0.79
Ziyang	0.360	0.368	0.359	0.364	0.372	0.366	0.356	0.352	0.363	0.352	0.342	0.359	−4.95
Kunming	0.674	0.658	0.671	0.663	0.676	0.666	0.628	0.606	0.602	0.587	0.579	0.637	−14.13
Qujing	0.404	0.381	0.411	0.399	0.385	0.370	0.364	0.356	0.359	0.361	0.352	0.377	−12.92
Yuxi	0.403	0.401	0.382	0.352	0.350	0.330	0.333	0.480	0.359	0.337	0.368	0.372	−8.69
Baoshan	0.358	0.347	0.346	0.328	0.340	0.327	0.324	0.323	0.347	0.332	0.345	0.338	−3.63
Zhaotong	0.356	0.331	0.329	0.298	0.315	0.325	0.352	0.332	0.372	0.374	0.342	0.339	−3.80
Lijiang	0.365	0.357	0.354	0.351	0.350	0.347	0.363	0.345	0.386	0.414	0.367	0.363	0.43
Pu′er	0.371	0.351	0.307	0.314	0.309	0.310	0.322	0.323	0.345	0.354	0.339	0.332	−8.60
Lincang	0.345	0.325	0.329	0.315	0.327	0.312	0.321	0.320	0.357	0.346	0.344	0.331	−0.37

**Figure 6:**
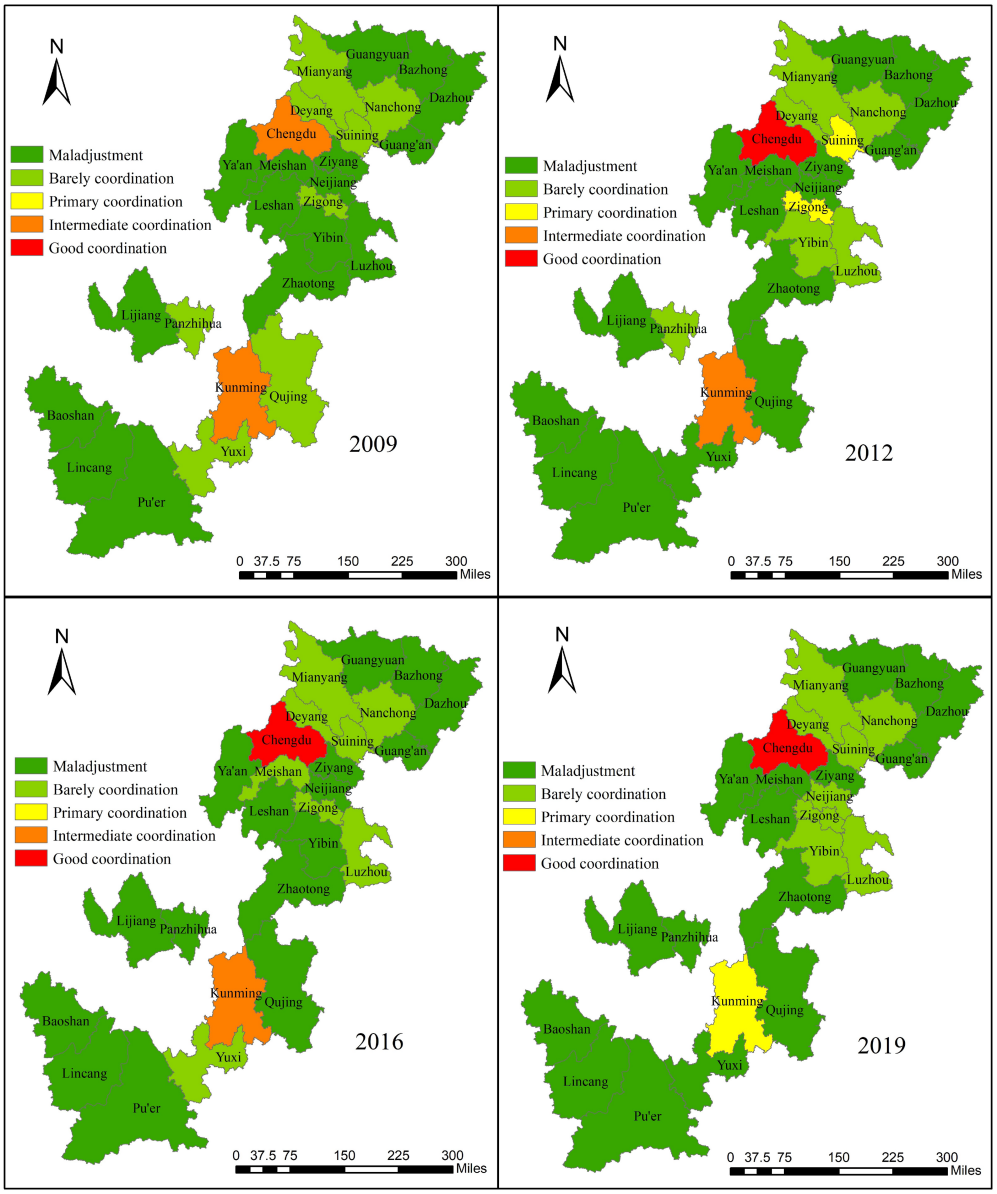
Spatial–temporal variation of coupling coordination degree.

According to ArcGIS10.8, to visualize the spatial differentiation of the CCD in 26 cities in Southwest China in 2009, 2012, 2016, and 2019. The spatial–temporal changes of the CCD are shown in Figure 6. In 2009, only Chengdu and Kunming were in the intermediate coordination level, while other cities were in the disordered state, and the overall CCD was not high, and no cities were in the primary coordination stage or good coordination stage, with large internal differences, indicating that the UR development and EE protection of all cities were in a relatively disharmony state during this period. From 2009 to 2012, five cities were promoted, two cities were decreased, and the remaining 19 cities remained unchanged. Zigong and Suining were promoted to the primary coordination stage, and the overall CCD level was improved. From 2012 to 2016, two cities improved, four cities decreased, 20 cities remained unchanged, and the overall level decreased slightly. From 2016 to 2019, three cities declined, two cities upgraded, and 21 cities remained unchanged, except for Kunming, which was degraded from intermediate coordination to primary coordination, and Chengdu, which remained in the good coordination stage. Although the overall CCD in 2019 improved to a certain extent compared with that in 2009, only Chengdu and Kunming reached coordination, and the scores of the CCD of the remaining cities were still in the disequilibrium stage below 0.5, with “strong core, weak middle, and edge” still prominent.

**Figure 7:**
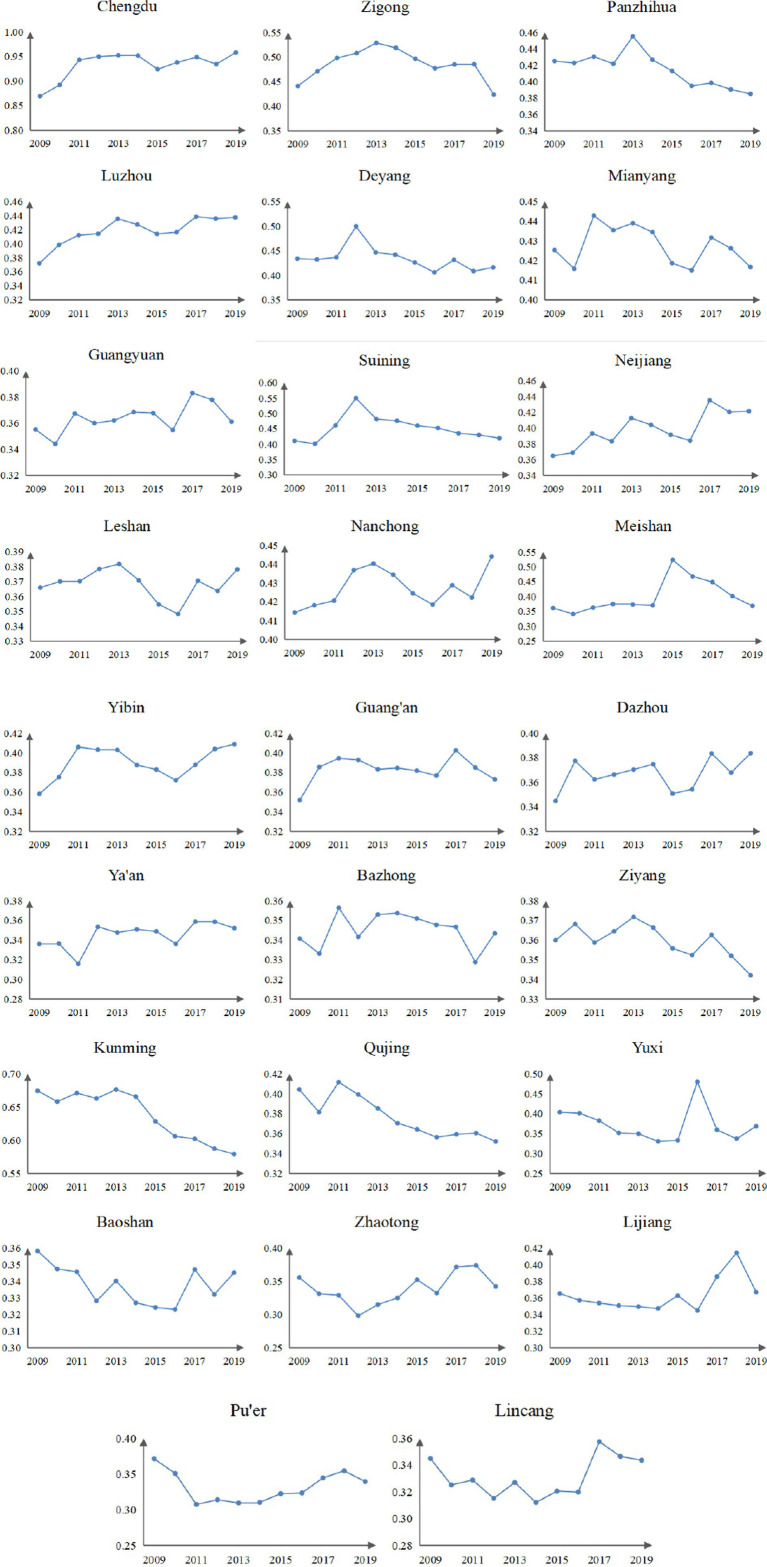
CCD evolution characteristics of 26 cities in Southwest China.

As for the temporal evolution of a single city, the trend evolution of a few cities is stable, and the fluctuation amplitude of most cities is obvious (Figure 7). Most of the cities with large changes show inverted “W“-shaped and inverted “V“-shaped time evolution characteristics, including Panzhihua, Deyang, Mianyang, Guangyuan, Suining, Meishan, Guang‘an, Ziyang, and Yuxi. It can be seen that these cities lack the impetus for economic growth and have failed to deal with the emission of pollutants caused by industrial development, which restricted the improvement of urban social welfare and the prevention ability of regional ecological risks and led to obvious fluctuations of CCD from 2009 to 2019 in these cities. How to optimize the industrial structure and how to improve the urban pollution control capacity is the key direction of urban transformation. It should be noted that Kunming, as the provincial capital, its CCD levels have shown a continuous decline from 2009 to 2019, which failed to grasp its good economic foundation and natural resource advantage.

### 3.4 The analysis of key obstacle factor diagnosis

#### 3.4.1 CCD obstacle degree identification results in 2009

**Figure 8:**
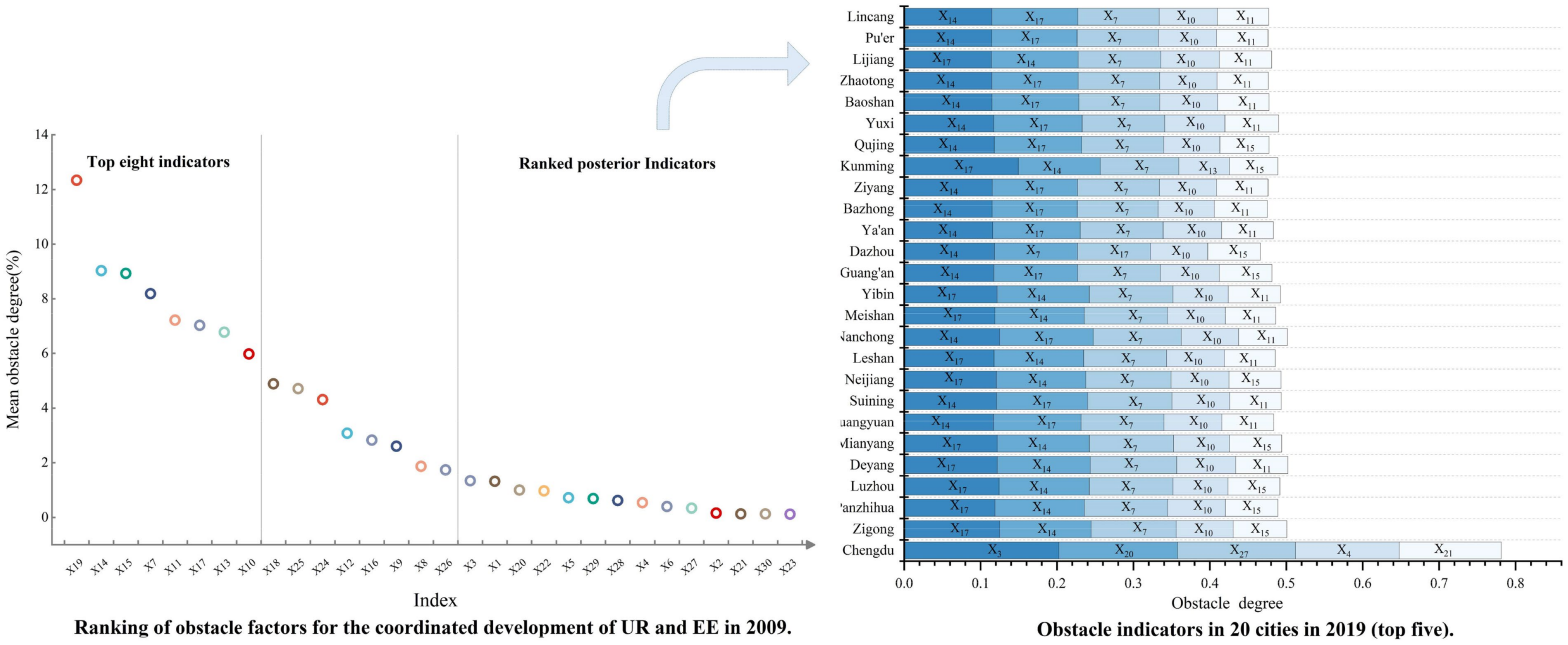
Obstacle factors for the CCD of UR and EE in 2009 and 2019.

From the perspective of a single index, the degree of obstacle varies greatly (Figure 8), and the average value is [0.12, 12.34%]. The top eight indexes with high obstacle degrees are, respectively, X_19_ (12.34%), X_14_ (9.03%), X_15_ (8.93%), X_7_ (8.19%), X_12_ (7.22%), X_17_ (7.03%), X_13_ (6.78%), and X_10_ (5.98%). From the system layer, the order of obstacle degree was social UR (46.04%) > spatial UR (23.05%) > economic UR (13.79%) > EE status (11.10%) > demographic UR (3.36%) > EE response (1.45%) > EE pressure (1.21%), indicating that social services for people’s livelihood are a major constraint on the level of coordination. Among them, in the social UR content, X_19_ (12.34%), X_14_ (9.03%), and X_15_ (8.93%) have greater barriers. In the early stage, the Southwest region had a small economic aggregate and poor infrastructure, which led to problems in the development of information technology, such as a large gap in the development of the Internet; the insufficient total volume of communication bodies; and small stock, low density, and poor connectivity of transportation infrastructure. These are some of the difficulties faced by urbanization. From the aspect of EE quality, EE status (11.10%) has the most prominent restricting effect, and the obstacle degree of X_25_ (4.71%) in EE level is the largest, showing that the shortage of green space resources and the irrational planning and utilization of land resources are still serious problems.

#### 3.4.2 CCD obstacle degree analysis in 2019

Compared with 2009, the overall characteristics showed little change (Table 5), and the average value is [0.06, 11.29%]. The top eight indexes with a high obstacle degree in 2019 are, respectively, X_17_ (11.29%), X_14_ (11.27%), X_7_ (10.44%), X_10_ (7.19%), X_11_ (6.32%), X_15_ (6.24%), X_13_ (5.89%), and X_24_ (4.93%). From the system layer, the order of obstacle degree was social UR (43.55%) > spatial UR (23.82%) > economic UR (15.90%) > EE status (10.88%) > demographic UR (4.20%) > EE pressure (1.03%) > EE response (0.62%), changed minorly from 2009. Social urbanization is still the most crucial element in curbing the increase in CCD. In the social UR level, X_17_ (11.29%) became the biggest limiting factor, indicating that there is still room for great progress in the continuous improvement of the public library facilities network system. From the aspect of EE quality, X_24_ (4.93%) instead of X_25_ (4.87%) became the biggest factor affecting the degree of obstacle, indicating that increasing urban green space is still an important construction direction. In terms of individual cities, with the exception of Chengdu, the major barriers in most cities are the same as the average. Chengdu has the largest obstacle degree at the demographic UR level, in which X_3_ (20.24%) is the biggest restricting factor, followed by the EE status level, in which industrial electricity consumption is the biggest restricting factor.

**Table 5:** System-level obstacles in the coordinated development of UR and EE in 2019.

City	UR subsystem				EE subsystem		
	Demographic (%)	Economy (%)	Space (%)	Society (%)	Pressure (%)	Status (%)	Response (%)
Chengdu	43.58	0.63	−0.01	15.53	16.68	16.82	6.77
Zigong	2.24	16.85	23.38	47.36	0.09	9.99	0.09
Panzhihua	1.75	14.52	25.00	45.92	1.20	10.29	1.32
Luzhou	2.87	16.59	23.85	46.27	0.45	9.85	0.13
Deyang	2.97	15.84	24.19	46.42	0.36	9.96	0.25
Mianyang	2.82	16.19	23.92	45.27	0.31	11.38	0.11
Guangyuan	2.51	16.54	25.52	44.35	0.09	10.82	0.16
Suining	2.49	18.08	24.29	46.22	0.09	8.60	0.22
Neijiang	2.65	18.61	24.62	43.57	0.53	9.88	0.14
Leshan	2.54	15.87	25.03	45.23	0.71	10.28	0.34
Nanchong	3.01	17.84	24.20	43.92	0.18	10.21	0.64
Meishan	2.47	17.44	24.87	44.79	0.23	10.14	0.07
Yibin	2.44	15.79	24.92	45.47	0.72	10.57	0.08
Guang’an	2.75	17.82	25.04	44.43	0.14	9.56	0.26
Dazhou	2.80	17.83	25.39	41.92	0.26	11.09	0.71
Ya’an	2.14	17.61	25.31	43.09	0.09	11.08	0.67
Bazhong	2.39	17.85	25.50	43.29	0.00	10.67	0.29
Ziyang	2.51	17.46	24.84	44.84	0.03	10.11	0.21
Kunming	2.78	13.02	21.94	46.92	1.66	12.57	1.11
Qujing	2.94	16.27	24.49	43.18	1.14	11.80	0.17
Yuxi	2.43	14.02	25.99	44.99	0.73	11.26	0.58
Baoshan	2.29	16.97	25.37	43.88	0.16	11.20	0.13
Zhaotong	2.86	16.41	25.15	43.06	0.37	11.64	0.51
Lijiang	2.93	15.16	25.56	45.08	0.08	11.01	0.19
Pu’er	3.14	15.82	25.45	43.67	0.40	10.99	0.54
Lincang	2.95	16.38	25.39	43.65	0.09	11.14	0.38

## 4 Discussion

**Figure 9:**
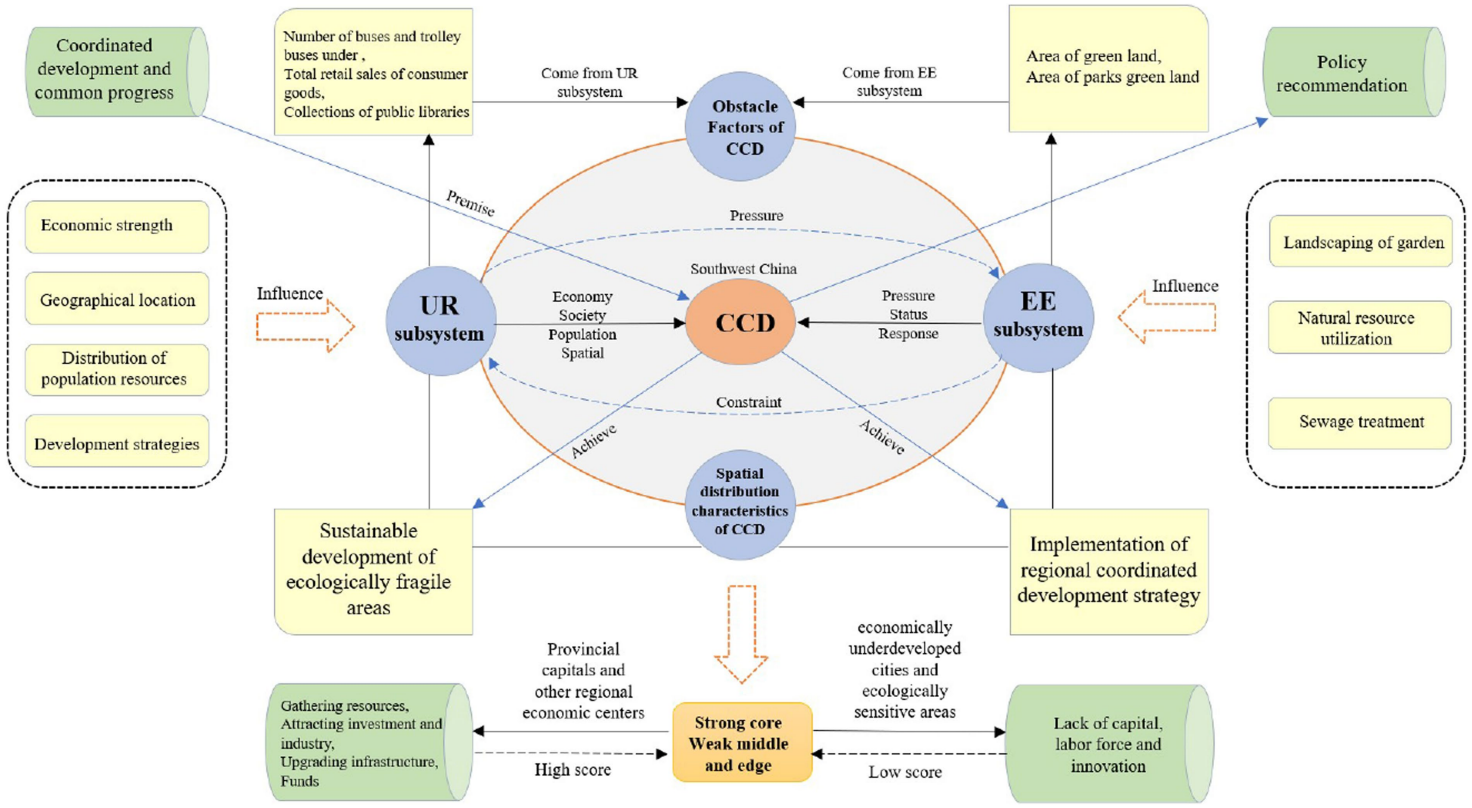
Conceptual framework for the CCD impact mechanism.

It is concluded from the CCD results that the CCD of 26 cities shows an obvious spatial imbalance. On the one hand, provincial capitals and other regional economic centers exhibit high CCD scores. They possess ample resources for resource aggregation, investment attraction, and industrial development. Furthermore, these centers allocate funds to address sustainability challenges, including pollution control and natural disaster management (56, 57). On the other hand, cities in economically underdeveloped and ecologically sensitive areas usually score low on CCD, and they generally lack capital, labor force, and innovation, which usually rely on backward industries with energy-extensive consumption and high emissions to keep the economy running, ignoring the oriented function of environmental management in industrial operation (58–60). The unilateral pursuit of interests often overlooks the imperative of EE protection, thereby impeding the symbiotic relationship between UR and EE (61, 62). Additionally, Luzhou and Kunming are both resource-based cities, and empirical findings indicate divergent trends in their CCD growth rates. Luzhou demonstrates a positive growth trajectory, whereas Kunming’s CCD shows a negative trend. This difference stems primarily from Kunming’s heavy reliance on environmental protection funds, coupled with inadequate investment in ecological technologies for ecological construction (63). There are many resource-based cities in Southwest China; however, intensive mining activities will lead to excessive consumption of resources in cities, resulting in soil erosion, land subsidence, biodiversity destruction, and groundwater pollution, and further widening the regional development gap (64). The conceptual framework for the CCD impact mechanism is shown in Figure 9.

The result of this study is consistent with the findings of Cai et al. (65), Li et al. (66), and Feng and Li (67), where the CCD between UR and EE varies considerably across regions at different stages of economic development. However, there are some studies that draw different conclusions. For example, Wu et al. (44) investigated urban agglomerations in northeastern China, revealing a consistent spatial pattern in the CCD of UR and EE systems. This pattern contrasts significantly with the findings presented in our study. The disparity can be attributed to the distinct economic development trajectories and ecological challenges between Northeast China and Southwest China. Specifically, Northeast China grapples with high levels of pollutant emissions as its primary environmental concern, coupled with relatively sluggish economic growth and minimal regional disparities, resulting in a stable spatial pattern. As discussed earlier, the prioritization of the ecological environment should not come at the expense of urbanization progress. Therefore, the insights gleaned from our study offer valuable guidance for ecologically fragile regions, akin to Northeast China, characterized by slower urbanization rates. In such areas, our findings advocate for prioritizing development initiatives, with a focus on fostering low-energy-consuming high-tech industries to propel regional economic advancement.

From the results of the obstacle factors model, the transition characteristics from 2009 to 2019 are characterized by two barrier factors: EE response and EE pressure. In 2009, EE response (1.45%) > EEE pressure (1.21%). In 2019, EE pressure (1.03%) > EE response (0.62%). From the contribution, both EE response and EE pressure are decreasing their hindering effect on CCD. However, the progress of EE response is in excess of EE pressure. It indicates that from 2009 to 2019, the government has taken vigorous measures to protect the environment, but more efforts need to be invested to truly alleviate environmental problems. In terms of specific indicators, the obstacle factor with the most significant contribution in 2009 is X19 (12.34%), and the most significant obstacle factor in 2019 is X17 (11.29%). The result reflects the progress of UR in China. In 2009, internet service users were still the most significant obstacle factor, and by 2019, its hindering effect on the CCD had been minimal. The 2009–2019 is the era of high-speed development of the internet and technology-driven, and the innovation has changed people’s lifestyles and facilitated the process of UR.

According to the obstacle factor analysis results in 2019, there are several obstacles that affect the CCD of UR and EE in Southwest China. In terms of UR quality, social UR has the greatest effect on CCD; among them, grassroots cultural services and travel convenience significantly have the strongest restraining effect on urban construction. The Southwest region has a remote geographical location, slow economic development, a relatively weak ability to absorb advanced technology and introduce advanced cultural systems and concepts, and public infrastructure, including cultural services and travel services, is still insufficient (68, 69). In addition, for the spatial UR subsystem layer, the obstacle degree of built-up area is the largest, mainly because the population explosion and the lack of living space seriously threaten the ecological quality of Southwest China (70). Due to topographic restrictions, most cities in Southwest China have limited space for construction. This persistent urban land structure deformity, coupled with the encroachment upon agricultural and ecological land, results in various ecological challenges such as inefficient land resource utilization, environmental pollution, and imbalanced urban–rural development (71, 72). Therefore, urban planning strategies in southwestern China must give due consideration to topographical constraints and transportation accessibility. In the policy system of UR, the social and cultural needs of the people should be met in addition to the pursuit of economic growth.

Economic urbanization is one of the main systems that hinder the development of CCD, among which the consumption level is the most restrictive degree by ensuring and building up people’s wellbeing. China’s Southwest cities are abundant in species and energy reserves and play a considerable role in ecological security, but they have fragile ecological environments, complex ecosystems, low productivity, and relatively backward socio-economic conditions, which leads to lagging economic development, insufficient consumer spending power, and affecting harmonious development with EE (73, 74). As the most widely distributed geomorphic region in China, the Southwest region has the characteristics of high sensitivity, low stability, and ecological vulnerability, which leads to serious land use problems, including soil erosion and rocky desertification in recent years, and has a serious inhibition effect on the promotion of greening in Southwest China (75, 76).

In terms of EE quality, EE status exerts the most significant influence on CCD. Among these factors, the reduction of green land poses the greatest hindrance to CCD enhancement. This finding aligns with the research conducted by Fu et al. (77). Vegetation coverage serves as a critical indicator for assessing EE. It not only regulates the dynamic balance of carbon budgets and mitigates the upward trend of greenhouse gases but also sustains biodiversity, serving as a vital component for achieving ecosystem resilience (78, 79). Furthermore, vegetation coverage plays a pivotal role in enhancing urban ecological environments, meeting recreational needs, shaping urban landscapes, beautifying surroundings, and mitigating disasters (80). Neglecting green space construction undermines ecological security, thereby disrupting the close relationship between UR and EE in Southwest China (81). Therefore, it is crucial for Southwest China to prioritize the preservation of adequate green space within its urban planning strategies. As part of the policy framework for ecological civilization construction, it is imperative to establish minimum requirements for key indicators such as vegetation coverage and park green space area.

## 5 Conclusion and policy implications

### 5.1 Conclusion

(1) From 2009 to 2019, the UR score of Southwest China showed an increasing trend, and the EE index showed a fluctuating downward trend. However, there were still obvious spatial and temporal differences. Due to the gap in economic strength, Chengdu and Kunming scored significantly higher than the other 24 cities, and it showed the spatial characteristics of Chengdu as the high-value center, followed by Kunming, while the average score of other cities hovered just approximately 0.1. Only six cities showed a trend of positive growth in EE quality.
(2) The urbanization level and EE quality of Southwest China show a relatively good coupling coordination relationship. The CCD level of most cities maintains a rapid upward trend, and the imbalance-type cities are significantly reduced and gradually improved. The spatial distribution still presents visible non-equilibrium traits between cities. Most cities have low CCD levels, showing a spatial distribution pattern with prominent characteristics of “strong core, weak middle, and edge.” In space, it gradually forms a development pattern with Chengdu as the core and Kunming as the small core area. The type of CCD is still mainly in the coordination stage.
(3) Obstacle analysis. In terms of the UR subsystem, the obstacle factors that affect CCD in Southwest China are mainly the number of buses and trolley buses under operation at year-end, the total retail sales of consumer goods, and collections of public libraries. In terms of the EE subsystem, the obstacle factors restricting the development of CCD are mainly areas of green land and areas of park green land, and this influencing factor shows a greater degree of inhibition than other factors.

### 5.2 Policy implications

(1) Tailor regional development strategies to local contexts. Our investigation reveals an imbalance in coupling coordination across cities in the Southwest region, with disparities between developed and less developed cities. To address this imbalance, cities in Southwest China should leverage their existing resource strengths and implement targeted measures to mitigate regional development gaps. For instance, Chengdu and Kunming can serve as focal points for regional development, acting as hubs to stimulate economic growth in surrounding non-provincial capital cities. This can be achieved by fostering the development of upstream and downstream industrial chains and establishing cross-regional cooperative organizations with robust feedback mechanisms. Additionally, efforts should be made to de-emphasize administrative divisions, encouraging deeper collaboration and innovation between cities.
(2) Adhering to the principle of people-centeredness and promoting the healthy and systematic development of UR. Our analysis identifies that grassroots cultural services and travel convenience significantly have the strongest restraining effect on urban construction. In response, the Southwest region should prioritize comprehensive coverage of social public services, local cultural amenities, and innovative urban and rural management practices. This includes enhancing transportation infrastructure, promoting the use of metro or light rail systems, and improving pedestrian and lane construction. Additionally, efforts should be made to elevate the cultural quality of the population by expanding access to public amenities such as libraries, museums, and cultural exhibition halls, ultimately aiming to benefit the populace. Simultaneously, it is essential not to constrain the urbanization process in the name of environmental protection. Encouraging rural-to-urban migration and steadily advancing reforms in urban settlement and household registration systems are crucial steps to foster sustainable urbanization.
(3) Enhance EE protection and restoration efforts. Our findings underscore the significance of preserving green spaces as the foremost factor impeding CCD. In response, cities in the Southwest region should prioritize urban green space planning and development. This includes expanding green spaces within communities, city parks, and other areas, as well as increasing the construction of wetlands, lakes, greenways, and ecological corridors. Moreover, strict adherence to arable land protection regulations is essential. Furthermore, initiatives should target key high-energy-consuming industries to promote the upgrade of pollution control equipment for smog, sulfur dioxide, and nitrogen oxide. Additionally, comprehensive measures should be taken to enhance the quality of the water environment. Emphasis should be placed on supporting the adoption of clean energy and renewable resources while fostering the cultivation and recruitment of personnel skilled in pollution control technology. Finally, the development of low-carbon, environmental, and green tourism sectors should be actively encouraged.

There are still a few limitations to this study that need further improvement and exploration. On the one hand, due to the workload of data collection in the construction of the index system, the selection of the number of indicators is relatively simple and insufficient, and some areas that are difficult to collect data are removed. In future research, it can be further enriched and supplemented to make the index system more representative and urban samples in the region more comprehensive. On the other hand, considering the importance of relevant indicators and in order to make the sample data as continuous as possible and avoid the sample size limitation, the missing values of part of the original data are supplemented, which may lead to a slight deviation between the data and the real value. Future data processing should reduce the probability of such cases. In further relevant research, the spatial scale can be expanded, and the research unit can be reduced from the municipal level to the district and county level, aiming to make the assessment more comprehensive and accurate. In addition, the new poverty phenomenon in the urbanization of ecologically fragile areas, the ecological poverty governance path, and the optimization of human–land coordination mode are important topics that need to be paid attention to. It will provide useful impetus to the exploration and study of the mechanisms of UR and EE and also to achieve the United Nations Sustainable Development Goals.

## Data availability statement

The data analyzed in this study is subject to the following licenses/restrictions: data is obtained from the corresponding author upon reasonable request. Requests to access these datasets should be directed to LL: 10001097@njtc.edu.cn.

## Author contributions

LL: Conceptualization, Funding acquisition, Investigation, Resources, Software, Writing – original draft, Writing – review & editing. YG: Formal analysis, Supervision, Validation, Visualization, Writing – original draft, Writing – review & editing. YL: Data curation, Formal analysis, Visualization, Writing – original draft. LZ: Writing – original draft, Writing – review & editing.
